# A Genome-Wide CRISPR/Cas9 Screen Reveals that Riboflavin Regulates Hydrogen Peroxide Entry into HAP1 Cells

**DOI:** 10.1128/mBio.01704-20

**Published:** 2020-08-11

**Authors:** Tamutenda Chidawanyika, Kenneth M. K. Mark, Surachai Supattapone

**Affiliations:** a Department of Biochemistry, Geisel School of Medicine at Dartmouth, Hanover, New Hampshire, USA; b Department of Medicine, Geisel School of Medicine at Dartmouth, Hanover, New Hampshire, USA; Washington University School of Medicine

**Keywords:** riboflavin, oxidative stress, hydrogen peroxide, aquaporin, KEAP1, riboflavin transporter, retinol saturase, P450 oxidoreductase, membrane transport

## Abstract

Extracellular hydrogen peroxide can induce oxidative stress, which can cause cell death if unresolved. However, the cellular mediators of H_2_O_2_-induced cell death are unknown. We determined that H_2_O_2_-induced cytotoxicity is an iron-dependent process in HAP1 cells and conducted a CRISPR/Cas9-based survival screen that identified four genes that mediate H_2_O_2_-induced cell death: *POR* (encoding cytochrome P450 oxidoreductase), *RETSAT* (retinol saturase), *KEAP1* (Kelch-like ECH-associated protein-1), and *SLC52A2* (riboflavin transporter). Among these genes, only *POR* also mediated methyl viologen dichloride hydrate (paraquat)-induced cell death. Because the identification of SLC52A2 as a mediator of H_2_O_2_ was both novel and unexpected, we performed additional experiments to characterize the specificity and mechanism of its effect. These experiments showed that paralogs of SLC52A2 with lower riboflavin affinities could not mediate H_2_O_2_-induced cell death and that riboflavin depletion protected HAP1 cells from H_2_O_2_ toxicity through a specific process that could not be rescued by other flavin compounds. Interestingly, riboflavin mediated cell death specifically by regulating H_2_O_2_ entry into HAP1 cells, likely through an aquaporin channel. Our study results reveal the general and specific effectors of iron-dependent H_2_O_2_-induced cell death and also show for the first time that a vitamin can regulate membrane transport.

## Abstract (precis)

Using a genetic screen, we discovered that riboflavin controls the entry of hydrogen peroxide into a white blood cell line. To our knowledge, this is the first report of a vitamin playing a role in controlling transport of a small molecule across the cell membrane.

## INTRODUCTION

Oxidative stress in cells occurs when the balance between reactive oxygen species (ROS) and the cellular antioxidant defense system is perturbed and the ROS levels increase beyond homeostatic concentrations (1, 2). ROS such as hydrogen peroxide (H_2_O_2_), superoxide radical (O_2_^•-^), and hydroxyl radical (OH^•^) are oxygen-containing molecules that are chemically reactive with other molecules in cells (3, 4). At physiologically optimal concentrations, ROS play important roles in signaling for proliferation, migration, and survival (5, 6). However, when ROS are generated rapidly or in large amounts to overwhelm the antioxidant response systems, unresolved oxidative stress damages macromolecules such as DNA, lipids, and proteins and leads to cell death through apoptotic or necrotic processes (7–10).

Different cell types, including leukocytes, platelets, epithelial cells, and endothelial cells, are programmed to generate and release high levels of ROS, specifically, H_2_O_2_, in response to bacterial infection, tissue injury, and chronic inflammation (4, 11–14). While this response occurs as part of a mechanism to alleviate the stress by killing invading bacteria or attracting leukocytes to the site of infection or injury, the exposure of cells in this environment to higher-than-normal concentrations of H_2_O_2_ can result in cell death (15). The combination of cell types in these environments is important for restoring homeostasis, but if homeostasis is not restored, the cells die (15). Despite extensive knowledge about the executors of apoptosis or necrosis as a result of high extracellular H_2_O_2_ concentrations (16, 17), the genes that mediate H_2_O_2_-induced cell death in these environments are unknown.

Consequently, we sought to identify the genes that mediate cell death in cells that are exposed to high concentrations of extracellular H_2_O_2_. We conducted a CRISPR/Cas9-based positive-selection screen in HAP1 cells using H_2_O_2_ as the selection tool in the screen. HAP1 cells are a near-haploid cell line that was derived from KBM7 cells, a leukemic cell line that was isolated from a patient in blast crisis (18). Since leukocytes often have to survive and function in environments of high extracellular H_2_O_2_ concentrations, HAP1 cells lent themselves appropriately to the goals of our screen. Our screen identified four novel genes that mediate H_2_O_2_-induced cell death in HAP1 cells. Further characterization of one of the genes from our screen revealed an unexpected regulatory role for riboflavin in the mediation of H_2_O_2_ entry into HAP1 cells.

## RESULTS

### Genes identified from a positive-selection CRISPR/Cas9 screen using H_2_O_2_.

**FIG 1:**
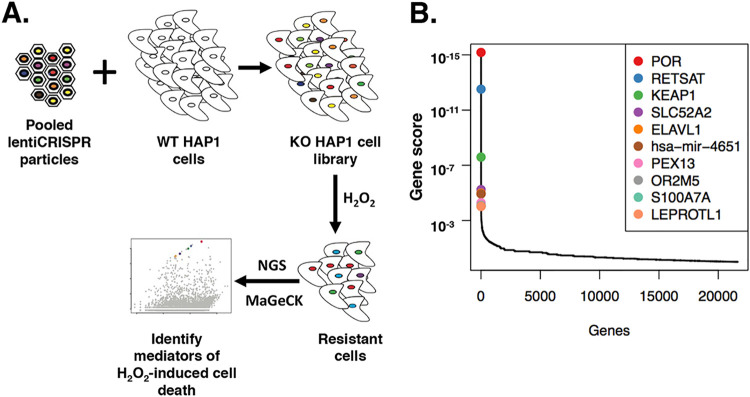
Genes that mediate H_2_O_2_-induced cell death isolated from a positive-selection screen in CRISPR/Cas9-modified HAP1 cells. (A) Positive-selection screen in CRISPR/Cas9-modified HAP1 cells using 350 μM H_2_O_2_. KO HAP1 cell libraries generated from WT HAP1 cells that were transduced with pooled lentiCRISPR libraries were treated with 350 μM H_2_O_2._ Next-generation sequencing (NGS) of the genomic DNA of resistant cells and MAGeCK bioinformatic analysis identified candidate genes. (B) Identification of top candidate genes that mediate H_2_O_2_-induced cell death in HAP1 cells through gene scores based on RRA *P* values generated from MAGeCK analysis.

To identify genes that mediate cell death induced by H_2_O_2_, a positive-selection screen was conducted in CRISPR/Cas9 knockout (KO) HAP1 cell libraries using H_2_O_2_ to induce oxidative stress and cell death (Fig. 1A). DNA from the surviving cells was isolated and deep sequenced to identify the genes represented in the enriched mutant populations. The screen identified 4 novel candidate genes as mediators of H_2_O_2_-induced cell death, namely, *POR*, *RETSAT*, *KEAP1*, and *SLC52A2* (Fig. 1B). *POR* encodes cytochrome P450 oxidoreductase, an endoplasmic reticulum membrane-bound protein that transfers electrons from NADPH to cytochrome P450s (19). *RETSAT* encodes retinol saturase, an NADH/NADPH- or FADH-dependent oxidoreductase that saturates all-*trans*-retinols to produce all-*trans*-dihydroretinoids and is primarily localized at the endoplasmic reticulum (20, 21). *KEAP1* encodes Kelch-like ECH-associated protein-1, a substrate adaptor that forms an E3 ubiquitin ligase complex with another protein to target Nrf2 for proteasomal degradation (22, 23). *SLC52A2* encodes solute carrier family 52 member 2, a riboflavin transporter localized to the plasma membrane (24, 25).

Using model-based analysis of genome-wide CRISPR/Cas9 knockout (MAGeCK), the strength of each candidate gene as a hit was determined by a combination of factors, namely, the number of good single guide RNAs (sgRNAs), the robust ranking aggregation (RRA) gene score, and the *P* value of the RRA gene score (26). Good sgRNAs included sgRNAs targeting a specific gene that showed enrichment in cells that were treated with H_2_O_2_ compared to untreated cells (26). The RRA gene score ranked candidate genes based on the degree of consistency seen in enrichment of multiple sgRNAs targeting a specific gene, and through determination of *P* values, significance scores were assigned for each gene (26). Lower RRA gene scores indicate genes of importance.

For *POR*, 5 of 6 sgRNAs were ranked as good sgRNAs and the gene had the lowest RRA score (6.71 × 10^−16^) (Fig. 1B; see also Table 1). For both *RETSAT* and *KEAP1*, 4 of 6 sgRNAs were ranked as good sgRNAs, with the genes having low RRA scores of 3.02 × 10^−13^ and 2.52 × 10^−8^, respectively (Fig. 1B; see also Table 1). *POR*, *RETSAT*, and *KEAP1* all shared the same low *P* value for their respective RRA gene scores (2.29 × 10^−7^). For *SLC52A2*, only 3 of the 6 sgRNAs that were designed to target *SLC52A2* were integrated into the genome of the HAP1 cells upon transduction and survived subsequent puromycin selection, and 2 of these 3 sgRNAs were ranked as good sgRNAs. The RRA gene score (5.78 × 10^−6^) for *SLC52A2* (Fig. 1B; see also Table 1) and its associated *P* value (1.67 × 10^−5^) were not as low as those of *POR*, *RETSAT*, and *KEAP1*.

**TABLE 1:** Ranking of genes in positive-selection H_2_O_2_ screen using MAGeCK

Rank	Gene	No. of sgRNAs^*a*^	RRA score^*b*^	*P*^*c*^	FDR^*d*^	No. of good sgRNAs	LFC^*e*^
1	POR	6	6.71E−16	2.29E−07	0.00165	5	10.263
2	RETSAT	6	3.02E−13	2.29E−07	0.00165	4	10.08
3	KEAP1	6	2.52E−08	2.29E−07	0.00165	4	3.0388
4	SLC52A2	3	5.78E−06	1.67E−05	0.090347	2	7.2286
5	ELAVL1	5	1.03E−05	2.26E−05	0.094884	2	−2.0804
6	hsa-mir-4651	2	1.19E−05	2.63E−05	0.094884	2	7.3621
7	PEX13	3	5.14E−05	0.00010454	0.323197	2	4.1784
8	OR2M5	6	7.87E−05	0.00017683	0.425193	2	−4.027
9	S100A7A	6	9.29E−05	0.00021114	0.456931	1	−3.6822
10	LEPROTL1	5	9.38E−05	0.00017317	0.425193	2	−1.0541

a The data in the “No. of sgRNAs” column refer to the number of sgRNAs targeting the gene.

b RRA score data were derived from a robust ranking aggregation (RRA) algorithm that determines the degree of consistency seen in the enrichment of multiple sgRNAs targeting a particular gene compared to sgRNAs targeting other genes.

c *P* values were generated for these RRA scores.

d FDR, false-discovery rate.

e LFC data represent log fold change of sgRNAs.

### *POR*, *RETSAT*, *KEAP1*, and *SLC52A2* mediate H_2_O_2_-induced cell death.

**FIG 2:**
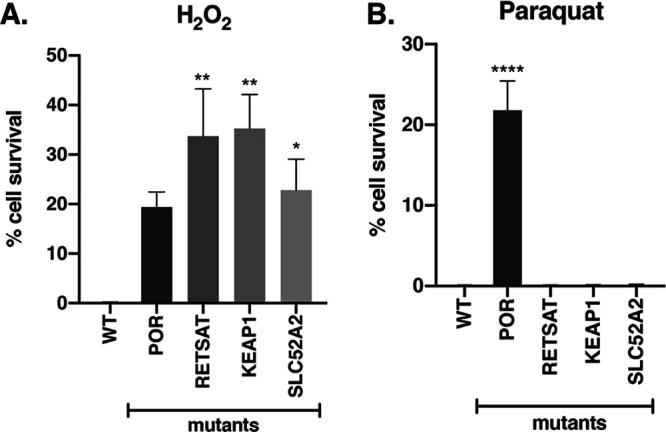
Cytotoxic response of knockout mutant cell lines to specific oxidative stress inducers. WT and knockout mutant monoclonal HAP1 cell lines were treated with (A) 350 μM H_2_O_2_ or (B) 375 μM paraquat and assessed for cell survival using trypan blue after 3 days of treatment. Error bars represent SEM (*n* = 6 independent experiments). *, *P < *0.05; **, *P < *0.005; ****, *P < *0.0001 (compared to WT cells).

To validate that *POR*, *RETSAT*, *KEAP1*, and *SLC52A2* mediate H_2_O_2_-induced cell death, knockout mutant cells of these genes were generated (see Fig. S2 in the supplemental material) and tested for resistance to H_2_O_2_-induced cytotoxicity. There was no significant difference in the baseline growth rates in the mutants compared to wild-type (WT) HAP1 cells (Fig. S3). Upon exposure to 350 μM H_2_O_2_ for 3 days, the *POR*, *RETSAT*, *KEAP1*, and *SLC52A2* mutants all conferred significant resistance to H_2_O_2_-induced cell death compared to WT HAP1 cells (Fig. 2A). Each of the knockout cell lines showed at least 20% cell survival after treatment with H_2_O_2_, while the control cell lines showed no cell survival. These findings show that *POR*, *RETSAT*, *KEAP1*, and *SLC52A2* mediated H_2_O_2_-induced cell death, at least in a subset of cells.

### *RETSAT*, *KEAP1*, and *SLC52A2* do not mediate paraquat-induced cell death, but *POR* does.

To determine if *POR*, *RETSAT*, *KEAP1*, and *SLC52A2* mediate cell death induced by different types of oxidative stress, we tested knockout mutant cell lines of these genes for resistance to a different ROS, the superoxide radical (O_2_^•-^), in comparison to H_2_O_2_. We used the herbicide methyl viologen dichloride hydrate (paraquat [PQ]) to generate O_2_^•-^ in the cells (27, 28). When the mutant cells were exposed to 375 μM paraquat, only *POR* mutant cells conferred significant resistance to paraquat-induced cell death. The *POR* mutant cells showed 20% cell survival after paraquat treatment, while the WT cells and the *RETSAT*, *KEAP1*, and *SLC52A2* mutant cells did not survive the treatment (Fig. 2B).

These data show that the roles of *RETSAT*, *KEAP1*, and *SLC52A2* in oxidative stress-induced cell death are specific to H_2_O_2_-induced cytotoxicity, while those of *POR* may be for general oxidative stress-induced cell death since *POR* mediated cell death induced by two ROS, H_2_O_2_, and O_2_^•-^ from paraquat.

### H_2_O_2_-induced cell death is caspase independent and iron dependent.

**FIG 3:**
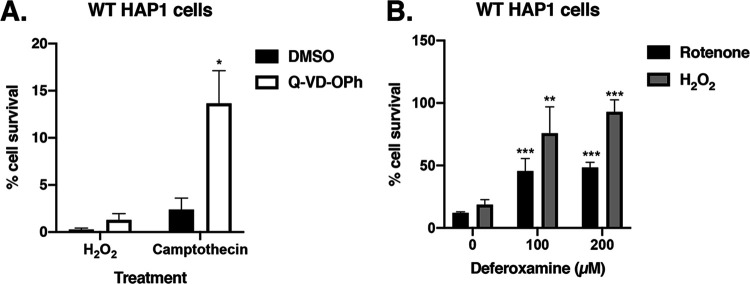
H_2_O_2_-induced cell death is caspase independent and iron dependent. (A) WT HAP1 cells were treated with 350 μM H_2_O_2_ or 5 μM camptothecin (CPT) in the presence of 200 μM Q-VD-OPh or DMSO. Cell survival was assessed after 3 days of treatment using trypan blue. Error bars represent SEM (*n* = 3 independent experiments). *, *P < *0.05 (compared to WT cells treated with DMSO). (B) WT HAP1 cells were treated with 350 μM H_2_O_2_ or 5 μM rotenone (RTN) in the presence of various concentrations of deferoxamine (an iron chelator), and cell survival (relative to control samples treated with the corresponding concentrations of deferoxamine without H_2_O_2_ or RTN) was assessed using trypan blue after a 24-h treatment. Error bars represent SEM (*n* = 3 independent experiments). **, *P < *0.004; ***, *P < *0.002 (compared to cells without deferoxamine).

Next, we wanted to determine the mechanism of H_2_O_2_-induced cell death in HAP1 cells. To determine if the mechanism was caspase dependent, we used Q-VD-OPh, a pan-caspase inhibitor, in conjunction with H_2_O_2_ to see if the Q-VD-OPh would rescue WT HAP1 cells from cell death. Camptothecin, which is known to kill cells in a caspase-dependent fashion, was used as a positive control (29, 30). The results show that Q-VD-OPh inhibited HAP1 cell death induced by camptothecin but not that induced by H_2_O_2_ (Fig. 3A). These data show that under our conditions, H_2_O_2_-induced cell death in HAP1 cells was caspase independent.

Through the iron-dependent Fenton reaction, H_2_O_2_ is converted to ROS, which can cause lipid peroxidation and subsequent cell death (31, 32). To determine if the H_2_O_2_-induced cell death seen under our conditions was iron dependent, we used deferoxamine, an iron chelator, to see if the removal of iron would rescue cells from H_2_O_2_-induced cell death. Rotenone, which has been shown to kill cells through an iron-dependent process, was used as a positive control (33). When WT HAP1 cells were treated with 350 μM H_2_O_2_ and 100 μM or 200 μM deferoxamine, cell survival increased significantly to greater than 50% compared to ∼20% survival in H_2_O_2_-treated cells without deferoxamine (Fig. 3B). Similarly, the same experiment performed with 5 μM rotenone instead of H_2_O_2_ showed that deferoxamine increased cell survival significantly to ∼40%, compared to ∼10% survival in rotenone-treated cells without deferoxamine (Fig. 3B). These data show that H_2_O_2_-induced cell death in HAP1 cells is iron dependent.

### SLC52A2 is the only riboflavin transporter that mediates H_2_O_2_-induced cell death.

**FIG 4:**
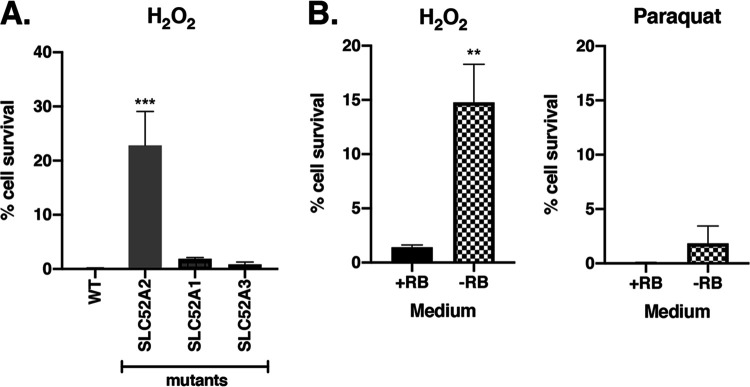
Riboflavin mediates cell death induced by H_2_O_2_ but not by paraquat. SLC52A1, SLC52A2, and SLC52A3 are all riboflavin transporters, with SLC52A2 having the highest affinity for riboflavin. (A) Knockout *SLC52A1*, *SLC52A2*, and *SLC52A3* monoclonal cells were grown in complete IMDM, treated with 350 μM H_2_O_2_, and assessed for cell survival using trypan blue after 3 days of treatment. Error bars represent SEM (*n* = 6 independent experiments). ***, *P < *0.0005 (compared to WT cells). (B) WT HAP1 cells were grown in base DMEMgfp-2 supplemented with riboflavin (+RB) or in base DMEMgfp-2 depleted of riboflavin (−RB) and were treated with 350 μM H_2_O_2_ (left panel) or 375 μM paraquat (right panel). After 3 days, cell survival was determined using trypan blue. Error bars represent SEM (*n* = 6 independent experiments). **, *P < *0.05 (compared to cells in riboflavin-containing medium [+RB]).

Since *POR*, *RETSAT*, and *KEAP1* have previously been found to be involved in mediating oxidative stress-induced cell death but *SLC52A2* has not (23, 34–36), we decided to focus the remainder of our experiments on dissecting how *SLC52A2* mediates H_2_O_2_-induced cell death in HAP1 cells. Among the members of a family of 3 riboflavin transporters, SLC52A2 has the highest affinity for riboflavin (25). To test whether the other family members, SLC52A1 and SLC52A3, might also play roles in H_2_O_2_-induced cell death, *SLC52A1*, *SLC52A2*, and *SLC52A3* knockout mutant cells were grown in complete Iscove's modified Dulbecco's medium (IMDM) and treated with 350 μM H_2_O_2_ for 3 days. Only the *SLC52A2* mutant cells conferred significant resistance to H_2_O_2_-induced cell death compared to WT cells, with greater than 20% of the cells surviving the H_2_O_2_ exposure (Fig. 4A). The WT and mutant *SLC52A1* and *SLC52A3* cells were unable to survive the H_2_O_2_ treatment (Fig. 4A), showing that SLC52A2 is the only riboflavin transporter that mediates H_2_O_2_-induced cell death in HAP1 cells.

### Riboflavin mediates cell death induced by H_2_O_2_ but not that induced by paraquat.

Since SLC52A2 has a known function in riboflavin transport, we wanted to determine whether SLC52A2 mediates H_2_O_2_-induced cell death through its function of riboflavin transport. WT HAP1 cells were grown in riboflavin-depleted (−RB) or riboflavin-containing (+RB) DMEMgfp-2 media and treated with 350 μM H_2_O_2_. After 3 days, only the cells in the riboflavin-depleted medium (−RB) showed a significantly higher percentage of surviving cells (∼15%) than was seen with the cells in media containing riboflavin (+RB), only about 2% of which survived (Fig. 4B). A similar experiment performed in *SLC52A2* KO cells in DMEMgfp-2 media reconstituted with riboflavin (+RB) showed ∼30% of cells surviving H_2_O_2_ treatment (Fig. S4A). These data show that the role of SLC52A2 in H_2_O_2_ cytotoxicity is based on its ability to transport riboflavin. Notably, they also reveal the importance of a single metabolite, riboflavin, in mediating H_2_O_2_-induced cytotoxicity.

To test whether riboflavin mediates cell death induced by other types of oxidative stress, WT HAP1 cells were exposed to the O_2_^•-^ generator paraquat in riboflavin-depleted or riboflavin-containing media. After 3 days, there were few cells surviving under either set of conditions, regardless of whether riboflavin was present (+RB) or not (−RB) (Fig. 4B). *SLC52A2* KO cells in riboflavin-containing medium were also unable to survive paraquat treatment (Fig. S4A).

Importantly, these data highlight the specific role that riboflavin plays as a mediator of H_2_O_2_-induced cell death in HAP1 cells. However, riboflavin does not mediate cell death induced by O_2_^•-^ as shown by the inability of a depletion of riboflavin to rescue WT cells from paraquat-induced cell death.

Riboflavin exposure to UV or visible light can result in the generation of cytotoxic products (37). To check if the oxidation of riboflavin by H_2_O_2_ was generating an artefactual toxic product that was the agent causing cell death in the WT HAP1 cells, we oxidized riboflavin through a pretreatment with H_2_O_2_ and then the mixture was treated with catalase to inactivate the H_2_O_2_. WT HAP1 cells were grown in riboflavin-containing or riboflavin-depleted media and treated with 350 μM H_2_O_2_ or oxidized riboflavin. As expected, the WT HAP1 cells that were grown in medium with riboflavin (+RB) did not survive the H_2_O_2_ treatment, while 50% of cells that were grown in riboflavin-depleted medium (−RB) showed a survival phenotype when treated with 350 μM H_2_O_2_ (Fig. S5). Treatment with oxidized riboflavin (RB_ox_) did not show a cytotoxic effect regardless of the media that the WT HAP1 cells were grown in (Fig. S5). In fact, the cells that were treated with RB_ox_ showed significant survival compared to the cells treated with H_2_O_2_. These data show that the riboflavin-dependent cytotoxicity that we observed in the presence of exogenously added H_2_O_2_ was not due to the formation of a toxic oxidized riboflavin adduct from H_2_O_2_ treatment but was more likely due to a biological interaction between riboflavin and HAP1 cells.

### Riboflavin regulates H_2_O_2_ entry into HAP1 cells.

**FIG 5:**
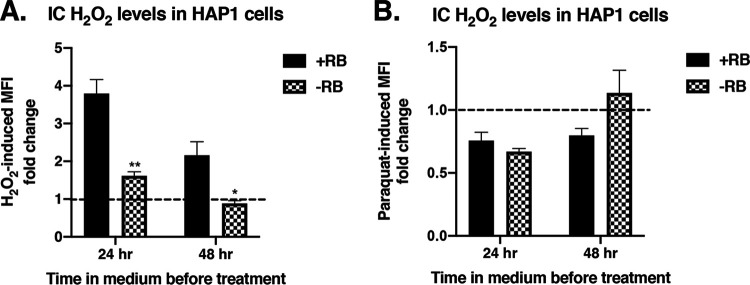
Effects of riboflavin on intracellular (IC) H_2_O_2_ in HAP1 cells in the presence of extracellular H_2_O_2_ or paraquat (PQ). HAP1 cells were grown in base DMEMgfp-2 supplemented with 1.063 μM riboflavin (+RB) or in base DMEMgfp-2 depleted of riboflavin (−RB) for 24 or 48 h. IC levels of H_2_O_2_ (determined by Hydrop MFI) in HAP1 cells were measured after exposure to exogenously added (A) 350 μM H_2_O_2_ or (B) 375 μM PQ. MFI fold change data shown represent MFI of H_2_O_2_-treated or PQ-treated cells relative to MFI of untreated cells. A fold change value of 1 (dotted line) represents no change. Error bars represent SEM (*n* = 3 independent experiments). *, *P = *0.0125; **, *P = *0.0014 (compared to cells in riboflavin-containing medium [+RB]).

Oxidative stress from high intracellular (IC) H_2_O_2_ levels can result in cell death (7–9), and our data show that riboflavin mediated H_2_O_2_-induced cell death (Fig. 4B). To determine if riboflavin affects IC H_2_O_2_ levels, we used a fluorescence-based assay to measure IC H_2_O_2_ levels in riboflavin-depleted cells treated with extracellular H_2_O_2_. These cells had been grown in the appropriate media for 24 or 48 h before treatment. Of note, IC H_2_O_2_ levels in riboflavin-containing (+RB) and riboflavin-depleted (−RB) WT HAP1 cells without any extracellular stimulation were similar (Fig. S6), showing that riboflavin did not influence baseline IC H_2_O_2_ metabolism or detection. Surprisingly, after riboflavin depletion in WT HAP1 cells, we found significantly greater increases in IC H_2_O_2_ levels in response to extracellular H_2_O_2_ in the presence of riboflavin (+RB) than in the riboflavin-depleted media (−RB) (Fig. 5A). A small (∼1.5-fold) H_2_O_2_-induced change in IC H2O_2_ levels was observed in riboflavin-depleted media (−RB) at 24 h, but not at 48 h, suggesting that endogenous riboflavin stores were more efficiently reduced by prolonged incubation in riboflavin-depleted media. A similar set of experiments in *SLC52A2* KO cells in media containing riboflavin (+RB) also did not show a significant change in IC H_2_O_2_ levels in response to extracellular H_2_O_2_ (Fig. S7A).

When paraquat was exogenously added to WT HAP1 (Fig. 5B) and *SLC52A2* KO (Fig. S7B) cells in the presence (+RB) or absence (−RB) of riboflavin, there were no significant differences in IC H_2_O_2_ levels, showing that the effect of riboflavin on IC H_2_O_2_ levels that we had observed was specific to extracellular H_2_O_2_. Taken together, these unexpected findings show that riboflavin plays an essential role in regulating H_2_O_2_ entry into HAP1 cells.

**FIG 6:**
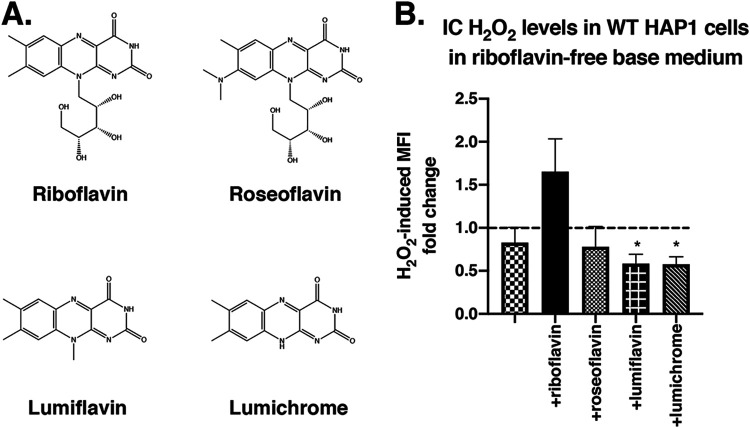
Mediation of H_2_O_2_ entry into HAP1 cells is specific to riboflavin compared to its structural analogs. (A) Chemical structures of riboflavin, roseoflavin, lumiflavin, and lumichrome. (B) HAP1 cells were grown in base DMEMgfp-2 for 24 h, followed by supplementation with 1.063 μM riboflavin (RB), roseoflavin (RF), lumiflavin (LF), or lumichrome (LC) for 16 h, as indicated. Intracellular (IC) levels of H_2_O_2_ (determined by Hydrop MFI) in HAP1 cells were measured after exposure to exogenously added 350 μM H_2_O_2_ or vehicle. MFI fold change data shown represent MFI of H_2_O_2_-treated cells relative to MFI of untreated cells. A fold change value of 1 (dotted line) represents no change. Error bars represent SEM (*n* = 6 independent experiments). *, *P < *0.05 (compared to cells in riboflavin-containing medium [+RB]).

To determine whether riboflavin’s effect on H_2_O_2_ cell entry is dependent on its chemical structure, we used roseoflavin, lumiflavin, and lumichrome, which are all structural analogs of riboflavin (38). HAP1 cells were grown in riboflavin-depleted medium for 24 h. Riboflavin, roseoflavin, lumiflavin, or lumichrome (Fig. 6A) was supplemented into the medium, and, after 16 h, cells were treated with Hydrop in the absence or presence of extracellular H_2_O_2_. The results show that only RB supplementation can mediate H_2_O_2_ entry into HAP1 cells since only the addition of RB can rescue the phenotype of an increase in IC H_2_O_2_ levels in the presence of extracellular H_2_O_2_ (Fig. 6B). Supplementation with the riboflavin analogs does not enable H_2_O_2_ entry into HAP1 cells; in fact, when RB is depleted from HAP1 cells for longer than 24 h, IC H_2_O_2_ levels decrease on exposure to extracellular H_2_O_2_.

**FIG 7:**
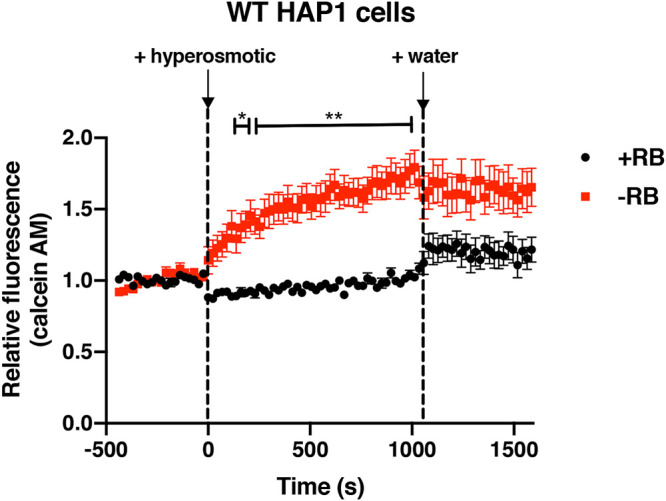
Effects of riboflavin on osmotic regulation in HAP1 cells. WT HAP1 cells were grown in base DMEMgfp-2 supplemented with 1.063 μM riboflavin (+RB) or in base DMEMgfp-2 without riboflavin (−RB) for 48 h. Relative fluorescence levels of calcein AM (a self-quenching dye) were used to determine volume changes upon addition of a hyperosmotic solution (2.5 M NaCl) to cells and then upon addition of water to cells. Error bars represent SEM (*n* = 12 independent experiments). Two-way ANOVAs were used to determine statistical differences between relative fluorescence values in samples (+RB or −RB) at different time points. *, *P < *0.05; **, *P < *0.01 (compared to +RB samples).

H_2_O_2_ entry into cells is known to be mediated by integral membrane proteins called aquaporins (AQPs), which also transport water into cells (39, 40). AQP function can be measured using volume-sensitive, self-quenching fluorescent dyes such as calcein AM in cells exposed to osmotically induced volume changes (41). To test whether riboflavin might control H_2_O_2_ entry by regulating AQP function, we then examined the effects of riboflavin depletion on volume changes in HAP1 cells exposed to osmotic stress. Upon addition of a hyperosmolar solution, HAP1 cells in riboflavin-containing media (+RB) immediately shrank as evidenced by decreased calcein fluorescence (Fig. 7, black circles, 0 s). On addition of water, the cells immediately regained volume as shown by the increase in fluorescence (Fig. 7, black circles, ∼1,050 s). Conversely, HAP1 cells grown in riboflavin-depleted medium (−RB) showed a paradoxical gradual increase in fluorescence upon treatment with a hyperosmolar solution (Fig. 7, red squares, between 0 and 900 s) and showed no change in fluorescence upon water addition (Fig. 7, red squares, ∼1,050 s). Overall, these data are consistent with the hypothesis that riboflavin mediates AQP function in HAP1 cells. The gradual increase in calcein fluorescence in riboflavin-depleted cells upon addition of a hyperosmolar solution was unexpected, but the fact that it occurred over several minutes suggests that it was not due to an osmotically induced volume change (since this should occur on a shorter time scale, e.g., within seconds, as seen in +RB cells).

To test whether riboflavin’s effect on H_2_O_2_ entry is cell type specific, we performed similar experiments to detect IC H_2_O_2_ in two epithelial cell lines, HEK293 cells and RPE-1 cells. In contrast to the observations made in WT HAP1 cells in the presence of extracellular H_2_O_2_, where IC H_2_O_2_ levels increased significantly in the presence of riboflavin compared to the levels measured in riboflavin-depleted medium, the IC H_2_O_2_ levels decreased in the presence of riboflavin in HEK293 and RPE-1 cells (Fig. S8). For both cell types, there was no difference between the decreases in IC H_2_O_2_ levels in riboflavin-containing and riboflavin-depleted medium, showing that, unlike in HAP1 cells, H_2_O_2_ entry into HEK293 and RPE-1 cells was not mediated by riboflavin; thus, H_2_O_2_ entry into these cells is not riboflavin dependent. These data suggest that the regulation of H_2_O_2_ entry into HAP1 cells is specific to this cell type and is likely to be associated with a HAP1-specific professional role.

## DISCUSSION

### *POR*, *RETSAT*, *KEAP1*, *SLC52A2*, and riboflavin mediate H_2_O_2_-induced cell death in HAP1 cells.

In this study, we sought to identify the genes that mediate cell death as a result of high extracellular H_2_O_2_ concentrations. We conducted a CRISPR/Cas9 survival screen in HAP1 cells, which are a human near-haploid leukemic cell line (42), using exposure to a high concentration of H_2_O_2_ as the selection tool for our screen. We determined that H_2_O_2_-induced cytotoxicity is an iron-dependent process in HAP1 cells and identified *POR*, *RETSAT*, *KEAP1*, and *SLC52A2* as mediators of H_2_O_2_-induced cell death. *POR*, *RETSAT*, *KEAP1*, and *SLC52A2* knockout mutants were resistant to a concentration of H_2_O_2_ that was lethal to WT cells, validating the screen results. Interestingly, none of the single knockout mutants conferred complete resistance to H_2_O_2_-induced cell death, suggesting that H_2_O_2_ may activate multiple cell death pathways simultaneously. Additionally, we found that *SLC52A2* mediates H_2_O_2_-induced cell death through its riboflavin transport function since HAP1 cells survived H_2_O_2_ treatment in the absence of riboflavin.

A few factors in our screening approach strengthened our study. First, the use of a CRISPR/Cas9-based knockout screen ensured that all of the genes identified in our screen are mediators of H_2_O_2_-induced cell death. Second, using a near-haploid cell line enabled efficient generation of complete gene knockouts in our library of the cells on which screens were performed. Last, the stringent conditions of the screen, in which less than 1% of the WT cells survived the H_2_O_2_ treatment, allowed us to be confident that survivors of our screen represented true hits. Another strength of our study is shown in the fact that one of the genes identified in our screen, *RETSAT*, was previously found to mediate H_2_O_2_-induced cell death (35), demonstrating the reliability of our CRISPR/Cas9 screening methodology. Our approach also had some limitations, however. It did not enable the identification of genes that are essential for cell viability or of redundant genes. The use of a polyploid cell line or of a CRISPR activation or inhibition screen would enable the identification of more mediators of H_2_O_2_-induced cell death. Our screen also did not enable the identification of genes that, on exposure to high concentrations of extracellular H_2_O_2_, do not cause cell death but instead modulate physiological processes in response to the exposure.

### Iron-dependent and caspase-independent H_2_O_2_-induced cell death in HAP1 cells.

We found that under our screening conditions, H_2_O_2_-induced cell death is iron dependent and caspase independent. Our findings are supported by previous studies of H_2_O_2_-induced cell death in dopaminergic SH-SY5Y neuroblastoma cells (43). Castino et al. showed that SH-SY5Y cells treated with 200 μM H_2_O_2_ die through an iron-dependent H_2_O_2_-induced cell death mechanism (43), as also seen in our experiments. In contrast to our findings, though, Yin et al. found that H_2_O_2_-induced cell death was caspase dependent in alveolar type II cells (44). The differences in our findings can be attributed to the fact that the studies were conducted in different cell types and that our concentration of H_2_O_2_ (350 μM) was significantly higher than the H_2_O_2_ concentration of 50 μM that was used in the studies employing alveolar type II cells.

Given that H_2_O_2_-induced cell death in our system was ROS dependent (from the exposure to H_2_O_2_), as well as iron dependent, it is possible that the mechanism of cell death in our cells is ferroptosis. From our results, we can infer that *POR*, *RETSAT*, *KEAP1*, and *SLC52A2* facilitate ferroptosis in HAP1 cells. *POR* has previously been found to mediate iron-dependent oxidative stress-induced cell death in a variety of cancer cells (45), while *KEAP1* may play a role in ferroptosis through inhibiting Nrf2 action since Nrf2 is a critical regulator of ferroptosis (46). *RETSAT* and *KEAP1* have been found to be a mediator of H_2_O_2_-induced cell death and a perturber of antioxidant cellular responses, respectively (23, 35). Further experimentation examining the different known key modulators of ferroptosis such as glutathione peroxidase 4 would be necessary to explore this idea (47).

### Riboflavin regulation of H_2_O_2_ entry into HAP1 cells.

Initially, the goal of our screen was to identify genes that are involved in H_2_O_2_-induced cell death. In addition to this knowledge, we discovered a riboflavin-dependent mechanism of H_2_O_2_ uptake in HAP1 cells through further exploration of *SLC52A2*, which encodes a riboflavin transporter.

Riboflavin (vitamin B2) is an essential vitamin for normal cellular function. Upon entry into cells, it is converted to flavin mononucleotide (FMN) and flavin adenine dinucleotide (FAD), which are important cofactors for flavoprotein function in redox biology and energy metabolism (38). Our data show that riboflavin’s mediation of cellular H_2_O_2_ import is specific to HAP1 cells. Interestingly, structural analogs of riboflavin, i.e., roseoflavin, lumiflavin, and lumichrome, were unable to phenocopy this effect of riboflavin, further highlighting the specificity of the riboflavin effect and also showing that the similarities in structure between these compounds are not responsible for the biological property of mediation of H_2_O_2_ import.

In contrast to our observations in HAP1 cells, riboflavin did not have an effect on H_2_O_2_ entry into two other cell lines, HEK293 or RPE-1 cells. These data emphasize the specificity of riboflavin regulation of H_2_O_2_ import in HAP1 cells and suggest that H_2_O_2_ transport control is a priority in HAP1 cells because of their professional function. It is entirely possible that since HAP1 cells are derived from a leukemic cell line, their behavior mimics that of leukocytes. In a riboflavin-deficient setting, leukocyte phagocytic function is decreased (48). Microbes that are phagocytosed by leukocytes are killed by the respiratory burst which can use H_2_O_2_ and other ROS to execute this function (49–51). It is possible that riboflavin allows leukocytes to assess their own ability to participate in the respiratory burst by giving an indication of cellular energy levels through FMN or FAD levels. Therefore, when riboflavin is abundant, H_2_O_2_ is readily transported from the extracellular space to assist in the respiratory burst. It is also possible that regulation of riboflavin levels could help protect certain cell types from autolysis (52).

It is worth considering the possibility that the H_2_O_2_-stimulated increase in IC H_2_O_2_ levels that we observed in HAP1 cells might be due to a change in IC H_2_O_2_ metabolism rather than to increased H_2_O_2_ transport across the plasma membrane. Although this explanation is formally possible, it does not seem likely because the putative metabolic change (i) would have to be stimulated by H_2_O_2_ but not paraquat; (ii) would have to occur in HAP1 cells, but not HEK293 or RPE-1 cells; and (iii) would have to be riboflavin dependent even though cells in untreated and riboflavin-depleted media have similar baseline IC H_2_O_2_ levels.

Since H_2_O_2_ transport across plasma membranes is known to be mediated by integral membrane proteins called aquaporins (AQPs) (39, 40), riboflavin may regulate H_2_O_2_ uptake directly or indirectly through an AQP in HAP1 cells. Our findings showing that volume regulation in HAP1 cells during osmotic stress depends on riboflavin support this idea. Direct mediation could involve interaction between riboflavin and the AQP, while indirect mediation could be based on intracellular FMN and FAD levels or on the presence of a flavoprotein that depends on FAD or FMN to interact with the AQP. In mammals, 4 of a family of 13 AQP isoforms are known to transport H_2_O_2_. For instance, AQP8 has been found to mediate H_2_O_2_ transport in leukemic cells (40, 53). Further studies will be needed to determine the details of the mechanism by which riboflavin mediates H_2_O_2_ entry into HAP1 cells.

### *RETSAT* and *KEAP1* mediation of cell death is specific to H_2_O_2_.

We found that *RETSAT* and *KEAP1* are specific in their mediation of H_2_O_2_-induced cell death since knockout mutants of these genes experienced paraquat-induced cell death. RETSAT was previously identified as a mediator of H_2_O_2_-induced cell death in NIH/3T3 cells, a mouse embryonic fibroblast cell line (35), as well as a generator of ROS in NIH/3T3 cells and in mice (36). KEAP1 represses antioxidant responses by targeting Nrf2, an important antioxidant transcription factor, for degradation (23). The identification of RETSAT and KEAP1 in our screen is therefore not surprising. However, our findings indicating that RETSAT and KEAP1 do not also mediate PQ-induced cell death are surprising, particularly with respect to KEAP1. Since Nrf2 is an important regulator of the antioxidant response in cells, we would expect that if KEAP1 mediates H_2_O_2_-induced cell death, it should also mediate PQ-induced cell death since both PQ and H_2_O_2_ cause oxidative stress. However, our data show that different ROS (H_2_O_2_ and O_2_^•-^ from PQ) require different mediators for cell death to occur. This may be due to their distinct mechanisms of toxicity. While PQ cytotoxicity occurs 48 h after exposure and relies on redox cycling for O_2_^•-^ generation (27, 28), H_2_O_2_ cytotoxicity occurs on a shorter time scale (within 24 h) and multiple cellular systems are affected (17, 54–56). It is possible that H_2_O_2_ treatment may generate a product that allows KEAP1 to trap Nrf2 in the presence of oxidative stress, while PQ treatment may not have the ability to generate the same or a similar product. These differences in the mechanisms of action could explain why only RETSAT and KEAP1 mediate H_2_O_2_-induced cell death.

Interestingly, only *POR*, which encodes an endoplasmic reticulum (ER)-localized enzyme, cytochrome P450 oxidoreductase (POR), mediated both H_2_O_2_ and PQ-induced cell death. POR was previously identified in a CRISPR/Cas9 positive-selection screen as a mediator of PQ-induced cell death through the generation of O_2_^•-^ in Jurkat cells (34). Our findings, in conjunction with the findings from the PQ screen, suggest that as an oxidoreductase, POR transfers electrons to different substrates and that this results in the generation of toxic ROS that result in cell death. This suggests a role for POR in the mediation of general oxidative stress-induced cell death.

### Conclusion.

**FIG 8:**
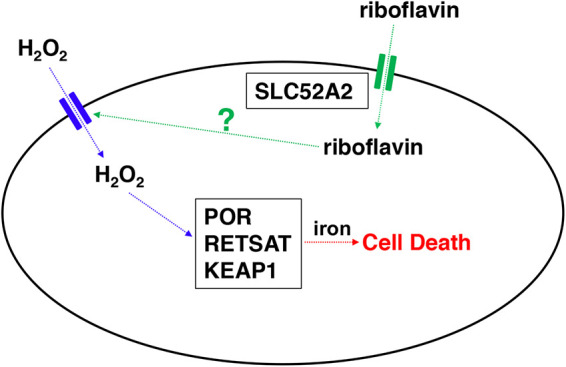
Potential model for H_2_O_2_-induced cell death in HAP1 cells. SLC52A2 transports riboflavin from the extracellular space into the cell. Riboflavin regulates H_2_O_2_ entry into the cell, likely through an aquaporin, where the putative aquaporin is shown in blue. POR, RETSAT, and KEAP1 then mediate iron-dependent, caspase-independent cell death.

In summary, we discovered that *RETSAT*, *KEAP1*, and *SLC52A2* are genes required specifically for H_2_O_2_-induced, iron-dependent cell death in HAP1 cells, whereas *POR* is a gene required for cell death induced by two different inducers of oxidative stress, i.e., H_2_O_2_ and paraquat. Further characterization of *SLC52A2* revealed a novel and specific role for riboflavin in the regulation of H_2_O_2_ entry into HAP1 cells, likely through an AQP. We propose a potential model for riboflavin-mediated H_2_O_2_-induced cell death in HAP1 cells (Fig. 8). To our knowledge, this is the first manuscript to report that a vitamin can specifically regulate membrane transport.

## MATERIALS AND METHODS

### Cell lines and vectors.

Materials were obtained from the following sources: WT HAP1 cells were from Horizon Discovery (Cambridge, United Kingdom); HEK293 cells were from ATCC (Manassas, VA, USA); and HEK293FT and RPE-1 cells were kindly provided by Michael Cole (Geisel School of Medicine at Dartmouth, Hanover, NH, USA) and Christopher Shoemaker (Geisel School of Medicine at Dartmouth, Hanover, NH, USA), respectively. A human GeCKO v2 library (1 plasmid system), lentiCRISPRv2, pMD2.G, and psPAX2 were obtained from Addgene (Cambridge, MA, USA).

### Cell culture and maintenance.

WT HAP1 cells were cultured in complete IMDM (MilliporeSigma, St. Louis, MO, USA), which consisted of IMDM supplemented with 10% fetal bovine serum (FBS) (HyClone, Logan, UT, USA), 4 mM l-glutamine, and 1× penicillin/streptomycin (Corning, Corning, NY, USA) unless otherwise specified. HEK293FT cells were cultured in IMDM supplemented with 10% FBS, 6 mM l-glutamine, 1× penicillin/streptomycin, and 1× nonessential amino acids (MilliporeSigma) for the first 24 h following thawing. After 24 h, G418 (MilliporeSigma) was added to the growth medium to reach a final concentration of 500 μg/ml. For experiments where riboflavin amount was adjusted, cells were maintained in base DMEMgfp-2 (riboflavin-depleted medium), which consisted of DMEMgfp-2 (Ervogen, Moscow, Russia) supplemented with 10% dialyzed FBS (Thermo Fisher Scientific, Waltham, MA, USA), 4 mM l-glutamine, 1× penicillin/streptomycin, and 19.45 μM pyridoxine hydrochloride (MilliporeSigma); and riboflavin (MilliporeSigma) was added at various concentrations as specified. Riboflavin depletion was performed for 24 or 48 h as specified. All cell lines were maintained at 37°C in 5% CO_2_. RPE-1 cells were cultured in DMEM:F12 (Lonza Biologics, Portsmouth, NH, USA) supplemented with 10% FBS, 0.01 mg/ml hygromycin B (Thermo Fisher Scientific), and 1× penicillin/streptomycin unless otherwise specified. HEK293 cells were cultured in DMEM (Corning) supplemented with 10% FBS, 4 mM l-glutamine, and 1× penicillin/streptomycin unless otherwise specified.

### Generation of CRISPR/Cas9 mutant libraries in HAP1 cells using GeCKO v2 plasmids.

GeCKO v2 libraries A and B were amplified and lentiviral particles were generated in HEK293FT cells as previously described (57). The 2 lentiviral sgRNA libraries (A and B) were designed to target 19,050 genes in total. Within the combined library, each gene was targeted by up to 6 unique sgRNAs. WT HAP1 cells were transduced with lentiviral particles at a multiplicity of infection (MOI) of 0.3 as previously described (57), and cells were grown under conditions of 1.5 μg/ml puromycin (MilliporeSigma) selection for 5 days with medium renewal every 48 h. Puromycin-selected cells were expanded and genomic DNA was extracted from 4 × 10^7^ cells using a blood and cell culture DNA midi kit (Qiagen, Germantown, MD, USA) to check the libraries for sgRNA representation through next-generation sequencing.

### Cytotoxicity assays for H_2_O_2_ in WT HAP1 cells.

WT HAP1 cells were seeded in 6-well plates (Corning) 24 h before stress induction. WT HAP1 cells at ∼60% confluence were treated with a range of concentrations of H_2_O_2_ (H325; Thermo Fisher Scientific). After 4 days, cell viability was determined using trypan blue (Corning) and a hemocytometer was used to count live cells. Cytotoxicity curves were generated for H_2_O_2_, and the concentration at which less than 1% of the HAP1 cells survived the H_2_O_2_-induced stress was determined (see Fig. S1 in the supplemental material). Data were plotted and analyzed using Prism software (GraphPad). As a result, 350 μM H_2_O_2_ was selected for screens in HAP1 CRISPR/Cas9 libraries.

10.1128/mBio.01704-20.1 FIG S1 Cytotoxicity curve for H_2_O_2_ in WT HAP1 cells. WT HAP1 cells were treated with H_2_O_2_ for 3 days to determine cytotoxic concentrations. Cell survival was determined using vital staining with trypan blue, and data are shown as percentages relative to untreated control cells. Download FIG S1, TIF file, 0.1 MB. Copyright © 2020 Chidawanyika et al. 2020 Chidawanyika et al. This content is distributed under the terms of the Creative Commons Attribution 4.0 International license.

10.1128/mBio.01704-20.2 FIG S2 Validation of mutant cell lines. Mutant monoclonal HAP1 cell lines were generated for the candidate genes from the H_2_O_2_ screen and from other cell lines that were used for comparison. The mutation status of cells was determined by Western blotting for the protein of interest or by genotyping the region of the gene that was targeted by an sgRNA for mutation in WT and mutant monoclonal HAP1 cells. (A to C) Western blots for (A) POR, (B) RETSAT, and (C) KEAP1 in WT and knockout mutant monoclonal HAP1 cell lines. (D) Genotyping results for *SLC52A1*, *SLC52A2*, and *SLC52A3* in WT and knockout mutant monoclonal HAP1 cell lines. Underlined regions indicate the location in the gene of interest that was targeted for mutation by an sgRNA (Table 2). Download FIG S2, TIF file, 0.2 MB. Copyright © 2020 Chidawanyika et al. 2020 Chidawanyika et al. This content is distributed under the terms of the Creative Commons Attribution 4.0 International license.

10.1128/mBio.01704-20.3 FIG S3 Cell viability of WT and mutant cells after 3 days of growth. WT and mutant cells were seeded in 12-well plates (1.5 × 10^6^ cells per well) and grown for 3 days. Cell viability was determined using trypan blue. Error bars represent SEM (*n* = 3 independent experiments). Download FIG S3, TIF file, 0.1 MB. Copyright © 2020 Chidawanyika et al. 2020 Chidawanyika et al. This content is distributed under the terms of the Creative Commons Attribution 4.0 International license.

10.1128/mBio.01704-20.4 FIG S4 Effects of riboflavin depletion on *SLC52A2* KO cells. WT and *SLC52A2* KO HAP1 cells were grown in base DMEMgfp-2 supplemented with 1.0623 μM riboflavin (+RB) or in base DMEMgfp-2 (−RB) for the indicated times and assessed for cell survival using trypan blue. (A) Cell survival of *SLC52A2* KO cells after a 24-h period of incubation in DMEMgfp-2 (+RB) medium, followed by 3 days of treatment in 350 μM H_2_O_2_ or 375 μM paraquat (PQ). Cell survival was determined using trypan blue. Error bars represent SEM (*n* = 6 independent experiments). *, *P = *0.0133 (compared to H_2_O_2_-treated cells). (B) Baseline viability of WT and *SLC52A2* KO cells after 48 h in +RB or −RB media. Error bars represent SEM (*n* = 6 independent experiments). ****, *P < *0.0001 (compared to cells in +RB medium). Download FIG S4, TIF file, 0.1 MB. Copyright © 2020 Chidawanyika et al. 2020 Chidawanyika et al. This content is distributed under the terms of the Creative Commons Attribution 4.0 International license.

10.1128/mBio.01704-20.5 FIG S5 Riboflavin oxidized by H_2_O_2_ is not the toxic agent in H_2_O_2_-induced cell death. WT HAP1 cells were grown in base DMEMgfp-2 supplemented with 1.063 μM riboflavin (+RB) or in base DMEMgfp-2 (−RB). WT HAP1 cells were treated with either 350 μM H_2_O_2_ or oxidized riboflavin (RB_ox_) to reach a final riboflavin concentration of 1.063 μM. After 3 days, cell survival was determined using trypan blue. Error bars represent SEM (*n* = 6 independent experiments). ***, *P < *0.0005 (compared to H_2_O_2_-treated cells). Download FIG S5, TIF file, 0.1 MB. Copyright © 2020 Chidawanyika et al. 2020 Chidawanyika et al. This content is distributed under the terms of the Creative Commons Attribution 4.0 International license.

10.1128/mBio.01704-20.6 FIG S6 Baseline intracellular (IC) H_2_O_2_ levels in HAP1 cells in the presence and absence of riboflavin. HAP1 cells were grown in base DMEMgfp-2 supplemented with riboflavin (+RB) or in base DMEMgfp-2 (−RB) for 24 or 48 h as indicated, and intracellular levels of H_2_O_2_ (baseline MFI determined by Hydrop MFI) in the cells were measured. Error bars represent SEM (*n* = 2 independent experiments). Download FIG S6, TIF file, 0.1 MB. Copyright © 2020 Chidawanyika et al. 2020 Chidawanyika et al. This content is distributed under the terms of the Creative Commons Attribution 4.0 International license.

10.1128/mBio.01704-20.7 FIG S7 Effects of riboflavin on entry of H_2_O_2_ into *SLC52A2* KO cells. *SLC52A2* KO cells were grown in base DMEMgfp-2 supplemented with riboflavin (+RB) for 24 or 48 h. IC levels of H_2_O_2_ (determined by Hydrop MFI) in cells were measured after exposure to exogenously added 350 μM H_2_O_2_ (A) or 375 μM paraquat (PQ) (B). MFI fold change data shown represent MFI of H_2_O_2_-treated or PQ-treated cells relative to MFI of untreated cells. A fold change value of 1 (dotted line) represents no change. Download FIG S7, TIF file, 0.1 MB. Copyright © 2020 Chidawanyika et al. 2020 Chidawanyika et al. This content is distributed under the terms of the Creative Commons Attribution 4.0 International license.

10.1128/mBio.01704-20.8 FIG S8 Effects of riboflavin on entry of H_2_O_2_ into HAP1, HEK293, and RPE-1 cells. HAP1, HEK293, and RPE-1 cells were grown in base DMEMgfp-2 supplemented with riboflavin (+RB) or in base DMEMgfp-2 (−RB). Intracellular levels of H_2_O_2_ (determined by Hydrop MFI) in the cell lines were measured after exposure of the cells to exogenously added 350 μM H_2_O_2_ or vehicle. MFI fold change data shown represent MFI of H_2_O_2_-treated cells relative to MFI of untreated cells. A fold change value of 1 (dotted line) represents no change. Download FIG S8, TIF file, 0.1 MB. Copyright © 2020 Chidawanyika et al. 2020 Chidawanyika et al. This content is distributed under the terms of the Creative Commons Attribution 4.0 International license.

### Positive-selection screen using H_2_O_2_ in HAP1 CRISPR/Cas9 libraries.

HAP1 CRISPR/Cas9 library cells (1.5 × 10^7^) were seeded on two 15-cm-diameter plates for each library (A and B libraries). Cells were treated 24 h later with medium containing 350 μM H_2_O_2_. Thereafter, cells were trypsinized and treated with medium containing fresh H_2_O_2_ (350 μM) every 3 days for a total of 12 days. Surviving cells were expanded in medium without H_2_O_2_ and genomic DNA was extracted from 4 × 10^7^ cells using a blood and cell culture DNA midi kit (Qiagen).

### Genomic DNA sequencing assayed via next-generation sequencing, data processing, and analysis.

To amplify sgRNA regions of interest in genomic DNA, 3 rounds of PCR were performed as previously described (57). The obtained samples were sequenced on a HiSeq 2500 Illumina platform. Model-based analysis of genome-wide CRISPR/Cas9 knockout (MAGeCK) was used to process and analyze data from the HiSeq sequencing platform (26). MAGeCK was run using default parameters with the human GeCKO v2 combined library (Addgene) as a reference. Fastq files for libraries A and B were combined and analyzed as a single data set. True gene hits from the MAGeCK results showed significant and consistent enrichment of two or more sgRNAs targeting the particular gene. Data files generated using MAGeCK are presented as supplemental data sets and represent the following: normalized counts (see Data Set S1 in the supplemental material) and a screen gene summary (Data Set S2). Table 1 shows positive-selection screen data derived from Data Set S2.

10.1128/mBio.01704-20.9 DATA SET S1 List of the number of normalized reads mapped to each sgRNA in the GeCKOv2 library for untreated control and H_2_O_2_-treated samples. The table represents the output generated using the ‘count’ command from MAGeCK after raw fastq files were trimmed to remove adapter sequences. Download Data Set S1, XLSX file, 3.8 MB. Copyright © 2020 Chidawanyika et al. 2020 Chidawanyika et al. This content is distributed under the terms of the Creative Commons Attribution 4.0 International license.

10.1128/mBio.01704-20.10 DATA SET S2 Ranking of genes in positive-selection H_2_O_2_ screen using MAGeCK. Gene-level enrichment was analyzed using the data in Data Set S1 by MAGeCK to determine if there if the levels of sgRNA abundance differed significantly between H_2_O_2_-treated and untreated cells. Ranking was determined by the *P* value calculated from a modified robust ranking aggregation (RRA) algorithm for a positive-selection screen. Download Data Set S2, XLSX file, 1.9 MB. Copyright © 2020 Chidawanyika et al. 2020 Chidawanyika et al. This content is distributed under the terms of the Creative Commons Attribution 4.0 International license.

### Phenotypic validations of candidate genes.

sgRNAs that showed the highest count numbers in our screens after MAGeCK analysis were used for validation (Table 2) (58). sgRNAs were cloned into the lentiCRISPRv2 backbone as previously described (58) to generate specific lentiCRISPR plasmids. Using the U6 forward primer (Table 2), the plasmids were subjected to Sanger sequencing to ensure that the sgRNAs were inserted in the lentiCRISPR plasmids correctly. Turbofectin 8.0 (Origene, Rockville, MD, USA) was used to transfect WT HAP1 cells with the lentiCRISPR plasmids per the manufacturer’s protocol to generate stable polyclonal mutant cell lines. Complete IMDM containing 1.5 μg/ml puromycin was used for a total of 5 days to select for transfected cells, with refreshment of puromycin every 48 h. Monoclonal cell lines were isolated using serial dilution in 96-well plates (Corning). For phenotypic validations, cell lines were seeded in 12-well plates (Corning), and 24 h later, at ∼60% confluence, the cells were treated with 350 μM H_2_O_2_ or 375 μM methyl viologen dichloride hydrate (paraquat) (MilliporeSigma). Cell viability was determined after 3 days using trypan blue and by counting live cells on a hemocytometer. Data were plotted and analyzed using Prism software (GraphPad).

**TABLE 2:** Sequences of primers and oligonucleotides

Identification	Sequence (5′→3′)
Sanger sequencing primer	
U6 forward primer	CGTGACGTAGAAAGTAATAATTTCTTGGG

sgRNA sequences targeting gene:	
POR	ACATGCCTCGCATCCCGTAG
RETSAT	TCAGCAGCCCACACCTGTCG
KEAP1	GCAATGAACACCATCCGAAG
SLC52A1	TGGTCACGGCACTGGTGAA
SLC52A2	AGCACCCACGCCCGCCCGTC
SLC52A3	CGGACGAGAAGAGCCGCAGC

Amplification primers for Sanger sequencing	
SLC52A1 forward primer	AGAAAGACCAACCAGCCCTG
SLC52A1 reverse primer	GCCTCCCCTCATACCTCTCT
SLC52A2 forward primer	GGCTCCACTCGCTCCTTC
SLC52A2 reverse primer	AATGCAACTGTCCTGCCACT
SLC52A3 forward primer	TCCCAGCTGTTTCCCTTCTA
SLC52A3 reverse primer	GGTGGGGAAGTCCTCATTG

### Immunoblotting.

Whole-cell lysates from WT and mutant monoclonal cell lines were isolated using PhosphoSafe extraction reagent (MilliporeSigma) per the manufacturer’s protocol. Lysates were run on 12% SDS polyacrylamide gels and visualized by Western blotting using the following antibodies: anti-cytochrome P450 reductase rabbit polyclonal antibody (ab13513) (Abcam, Cambridge, MA, USA) (1:1,000 dilution in 5% [wt/vol] milk–TBST [Tris-buffered saline with Tween 20]), RETSAT rabbit polyclonal antibody (PA565443) (Thermo Fisher Scientific) (1:1,000 dilution in 5% [wt/vol] bovine serum albumin [BSA]–TBST), KEAP1 (D6B12) rabbit monoclonal antibody (catalog no. 8047) (Cell Signaling Technology, Danvers, MA, USA) (1:1,000 dilution in 5% [wt/vol] BSA–TBST), and anti-rabbit IgG horseradish peroxidase (HRP)-linked antibody (catalog no. 7074) (Cell Signaling Technology, Danvers, MA, USA) (1:2,000 dilution in 5% [wt/vol] milk–TBST). All primary antibody incubations were performed at 4°C overnight, and all secondary antibody incubations were performed at room temperature for 1 h. Western blots were processed using SuperSignal West Femto maximum sensitivity substrate (Thermo Fisher Scientific). Western blotting performed for POR, RETSAT, and KEAP1 showed that these proteins were all expressed in the WT HAP1 cells but that they were not expressed in the isolated monoclonal mutant cell lines, as indicated by the absence of a protein band at the appropriate molecular weight in the mutant cells (Fig. S2A, B, and C).

### Genotyping of monoclonal cell lines.

Genomic DNA was isolated from WT and mutant monoclonal HAP1 cell lines using Quick Extract DNA extraction solution (Lucigen, Middleton, WI) per the manufacturer’s protocol. Primers (Table 2) were designed to amplify regions of interest in the *SLC52A1*, *SLC52A2*, and *SLC52A3* genes where CRISPR/Cas9 modifications were likely to have occurred based on the sgRNA sequences used to generate the cell lines. Kapa HiFi HotStart DNA polymerase (Kapa Biosystems, Cape Town, South Africa) was used to generate amplicons that were analyzed for correct size on 1% Omnipur agarose gels (MilliporeSigma). Amplicons were subjected to gel extraction using a gel extraction kit (Qiagen) and were subjected to Sanger sequencing using the appropriate *SLC52A1*, *SLC52A2*, and *SLC52A3* sequencing primers (Table 2) to determine the nature of the mutations in the cell lines (Fig. S2D). All mutations resulted in frameshifts and early stop codons that generated knockouts of the targeted genes.

### Cytotoxicity assays for H_2_O_2_ with caspase inhibition and iron chelation.

WT HAP1 cells (1.5 × 10^5^ cells per well) were seeded in 12-well plates and incubated for 24 h at 37°C in 5% CO_2_. For the caspase inhibitor assays, a pan-caspase inhibitor, Q-VD-OPh (ApexBio, Boston, MA, USA) was used at a stock concentration of 200 mM in DMSO (dimethyl sulfoxide). For iron chelation assays, deferoxamine (MilliporeSigma) was used at a stock concentration of 100 mM in water. For caspase inhibition assays, cells in each well were treated with 500 μl of complete IMDM with 400 μM Q-VD-OPh or DMSO (1 μl per well, which is equal in volume to the added Q-VD-OPh) for 1 h. For the iron chelation assays, the cells in each well were treated with 500 μl of complete IMDM with 200 μM or 400 μM deferoxamine for 1 h. a 500-μl volume of complete IMDM containing 700 μM H_2_O_2_ was added such that the final treatment concentrations per well in the caspase inhibition assay were 200 μM Q-VD-OPh and 350 μM H_2_O_2_, and the final treatment concentrations per well in the iron chelation assay were 100 μM or 200 μM deferoxamine and 350 μM H_2_O_2_. After 3 days of treatment for the caspase inhibitor assay and a 24-h treatment for the iron chelation assay, cell viability was determined using trypan blue and a hemocytometer. Cell survival was expressed as the percentage of the surviving cells in wells that were treated with Q-VD-OPh or deferoxamine and H_2_O_2_ relative to cells treated with Q-VD-OPh or deferoxamine only. Data were plotted and analyzed using Prism software.

### Cytotoxicity assays in riboflavin-depleted (−RB) and riboflavin-containing (+RB) media.

WT HAP1 or *SLC52A2* KO cells (1.5 × 10^5^ cells per well) were seeded and grown in 12-well plates for 48 h at 37°C in 5% CO_2_ in base DMEMgfp-2 (−RB) or in base DMEMgfp-2 supplemented with 1.063 μM riboflavin (+RB). Cell viability was determined using trypan blue. *SLC52A2* KO cells showed a significant decrease in cell viability in riboflavin-depleted medium (−RB) compared to cells in riboflavin-containing medium (Fig. S4B). This result indicates that neither riboflavin depletion for 48 h nor *SLC52A2* knockout alone is sufficient to fully deplete endogenous riboflavin stores and that full depletion of cellular riboflavin is lethal to HAP1 cells. Therefore, all cytotoxicity assays in *SLC52A2* KO cells were conducted only in riboflavin-containing medium (+RB) to ensure that cell viability was not compromised. WT HAP1 or *SLC52A2* KO cells (1.5 × 10^5^ cells per well) were seeded in 12-well plates for 24 or 48 h at 37°C in 5% CO_2_ in base DMEMgfp-2 (−RB) or in base DMEMgfp-2 supplemented with 1.063 μM riboflavin (+RB). Cells were treated with 350 μM or 375 μM paraquat for 3 days, and cell viability was determined using trypan blue staining.

### Cytotoxicity of riboflavin oxidized by H_2_O_2_.

Riboflavin was dissolved in water and then treated with 350 μM H_2_O_2_ for 1 h at 37°C. Catalase (MilliporeSigma) was dissolved in 50 mM potassium phosphate buffer to a stock concentration of 10 mg/ml. To stop the oxidation of riboflavin, catalase was added to the riboflavin to reach a final concentration of 5 mg/ml and the resulting mixture was incubated at 25°C for 20 min. This product was oxidized riboflavin (RB_ox_). The generated riboflavin product was added to WT HAP1 cells at ∼60% confluence to reach a final riboflavin concentration of 1.063 μM. The cells had been grown either in base DMEMgfp-2 for 24 h or in base DMEMgfp-2 supplemented with 1.063 μM riboflavin. WT HAP1 cells in both sets of media were also treated with 350 μM H_2_O_2_ as a control.

### Intracellular (IC) H_2_O_2_ detection using Hydrop and flow cytometry.

WT HAP1 cells were maintained in base DMEMgfp-2 for 24 or 48 h as indicated. For experiments with flavin analogs, 1.063 μM riboflavin, roseoflavin, lumiflavin, or lumichrome (Cayman Chemical Company, Ann Arbor, MI, USA) was added to the base medium. Experiments with *SLC52A2* KO cells were performed for 24 and 48 h in base DMEMgfp-2 supplemented with 1.063 μM riboflavin as indicated to preserve cell viability. For experiments with paraquat, after 24 or 48 h of maintenance in medium, cells were treated in medium containing 375 μM paraquat or vehicle for a further 40 h. Cells were stained in 1 μM Hydrop (Goryo Chemical, Sapporo, Japan) in the appropriate growth medium (with or without 350 μM H_2_O_2_ or 375 μM paraquat) for 1 h at 37°C in 5% CO_2_. Cells were washed once in phosphate-buffered saline (PBS) and resuspended in PBS with or without 350 μM H_2_O_2_ or 375 μM paraquat. The cells were filtered through 40-μm-pore-size cell strainers. Fluorescence was detected using a MACSQuant VYB flow cytometer at an excitation wavelength of 488 nm. For each experimental sample, 50,000 events were collected. FlowLogic software (Inivai Technologies) was used to visualize and collect median fluorescence intensity (MFI) data. Hydrop MFI was the fluorescent indicator for intracellular H_2_O_2_. MFI data were background corrected according to the MFI of unstained, untreated cells using subtraction. MFI fold change was calculated as the MFI of a H_2_O_2_-treated or paraquat-treated sample relative to the MFI of an untreated sample. Data were plotted and analyzed using Prism software (GraphPad).

### Measurement of volume changes using calcein AM.

WT HAP1 cells were seeded at 3 × 10^4^ cells per well in 200 μl base DMEM-gfp2 and at 1.5 × 10^4^ cells per well in 200 μl base DMEM-gfp supplemented with 1.063 μM riboflavin in a 96-well black-walled plate (catalog no. 137101; Nunc, Thermo Fisher Scientific). After a 48-h incubation, cells were stained in 50 μl of a solution of 10 μM calcein AM (Thermo Fisher Scientific) with DMEM-gfp2 supplemented with 10% dialyzed FBS. Each stained well was paired to a control well, to which 50 μl of DMEM-gfp2 supplemented with 10% dialyzed FBS was added. Cells were stained for 45 min at 37°C in 5% CO_2_, and then the staining medium was removed from the cells. Cells were washed once in 100 μl PBS, and 50 μl of PBS was added to each well. The 96-well plate was transferred at 37°C to a plate reader, where fluorescence of calcein AM was excited at 490 nm and detected at 520 nm. Calcein AM is a self-quenching dye; therefore, as the intracellular concentration of the dye increases, a decrease in fluorescence is expected (59, 60). Baseline fluorescence measurements were measured for 6 min, and then measurements were stopped to add 50 μl of 2.5 M NaCl to all wells (hyperosmotic solution). The 96-well plate was returned to the plate reader, and fluorescence measurements were collected for 15 min after a 10-s orbital shake. After 15 min, measurements were stopped and 100 μl of molecular-grade water (Corning) was added to all of the wells. Fluorescence measurements were collected for 10 min. SoftMax Pro software (Molecular Devices, San Jose, CA, USA) was used to collect the fluorescence data. Fluorescence measurements taken after treatments with the hyperosmolar solution or water for each well were normalized to the average of the baseline fluorescence measurements performed for the same well. Fluorescence measurements in stained wells were then normalized to the measurements in the control wells to which they were paired in order to calculate relative fluorescence levels of calcein AM as an indicator of cell volume. Data were plotted using Prism software.

### Statistical analysis.

Data are presented as means ± standard errors of the means (SEM). Statistical significance was determined using one-way analysis of variance (ANOVA) or Student's *t* test (GraphPad Prism software) as appropriate for each figure. Statistical significance was determined at the 0.05 level, and a normal distribution of the data was assumed.
