# Supplementary figures and images for: A Genome-Wide CRISPR/Cas9 Screen Reveals that Riboflavin Regulates Hydrogen Peroxide Entry into HAP1 Cells
Source: mBio. 2020 Aug 11;11(4):e01704-20. doi: 10.1128/mBio.01704-20 (PMC7439486; doi:10.1128/mBio.01704-20)

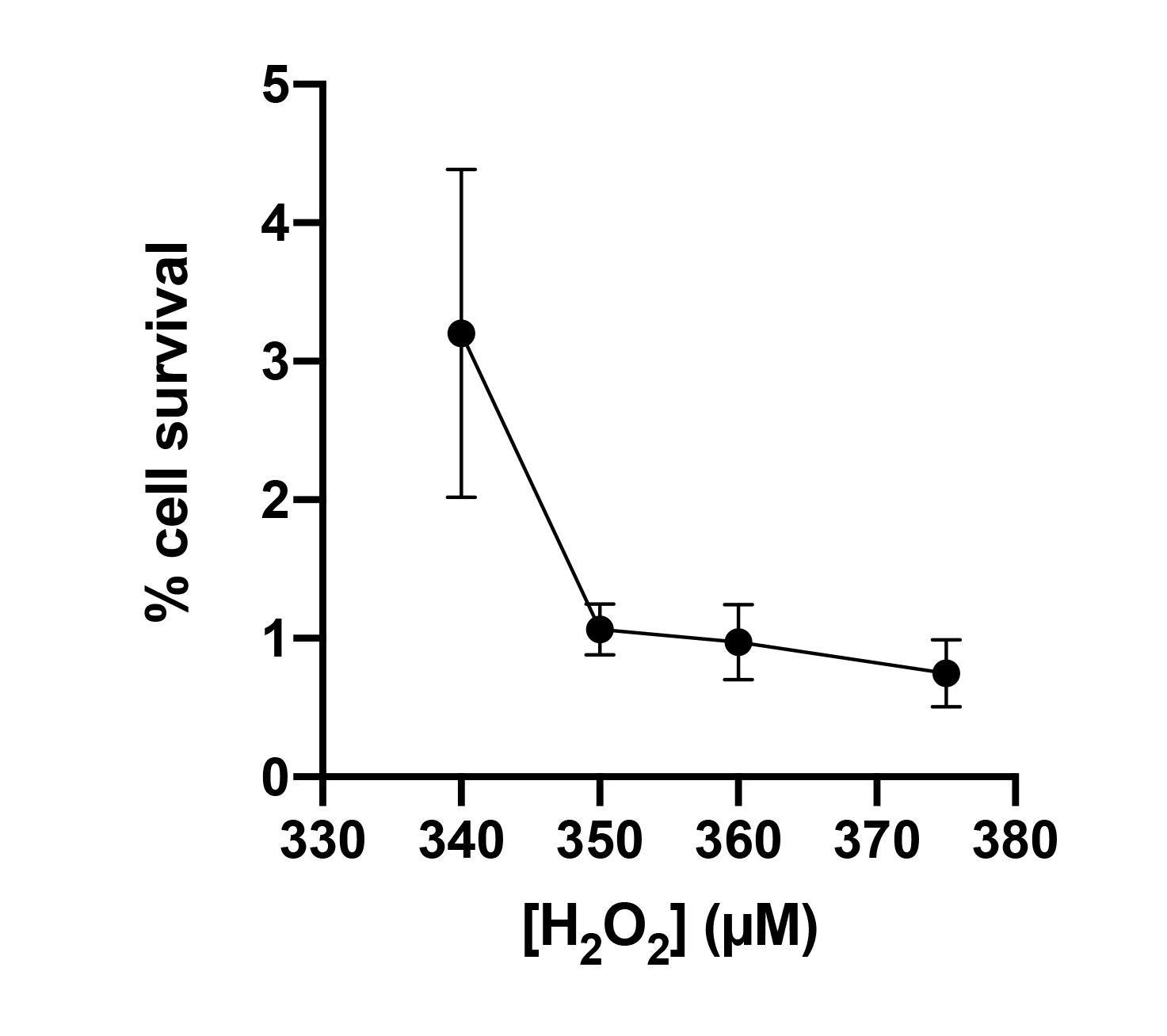

Supplement: FIG S1 [file mBio.01704-20-sf001.tif]

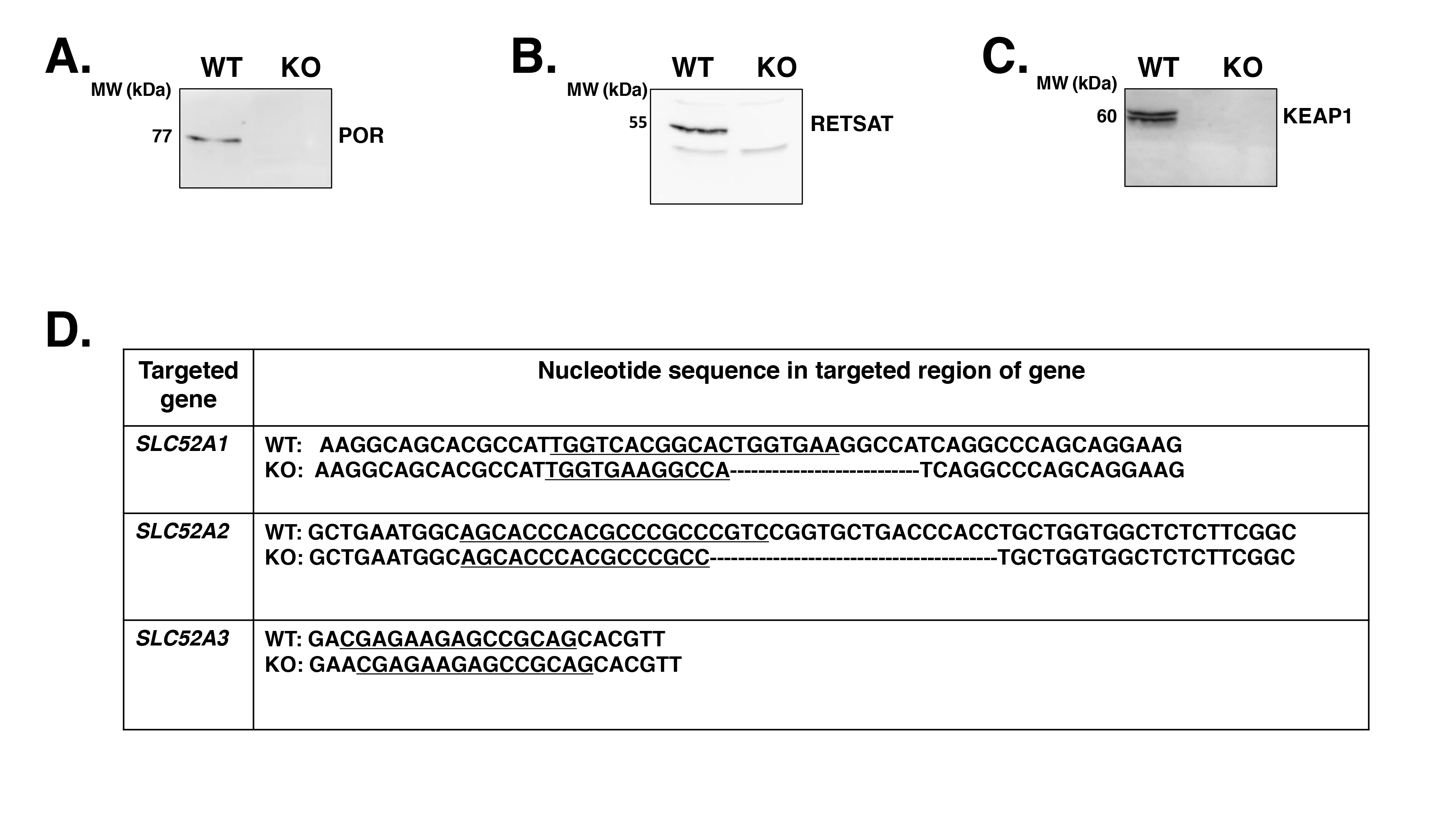

Supplement: FIG S2 [file mBio.01704-20-sf002.tif]

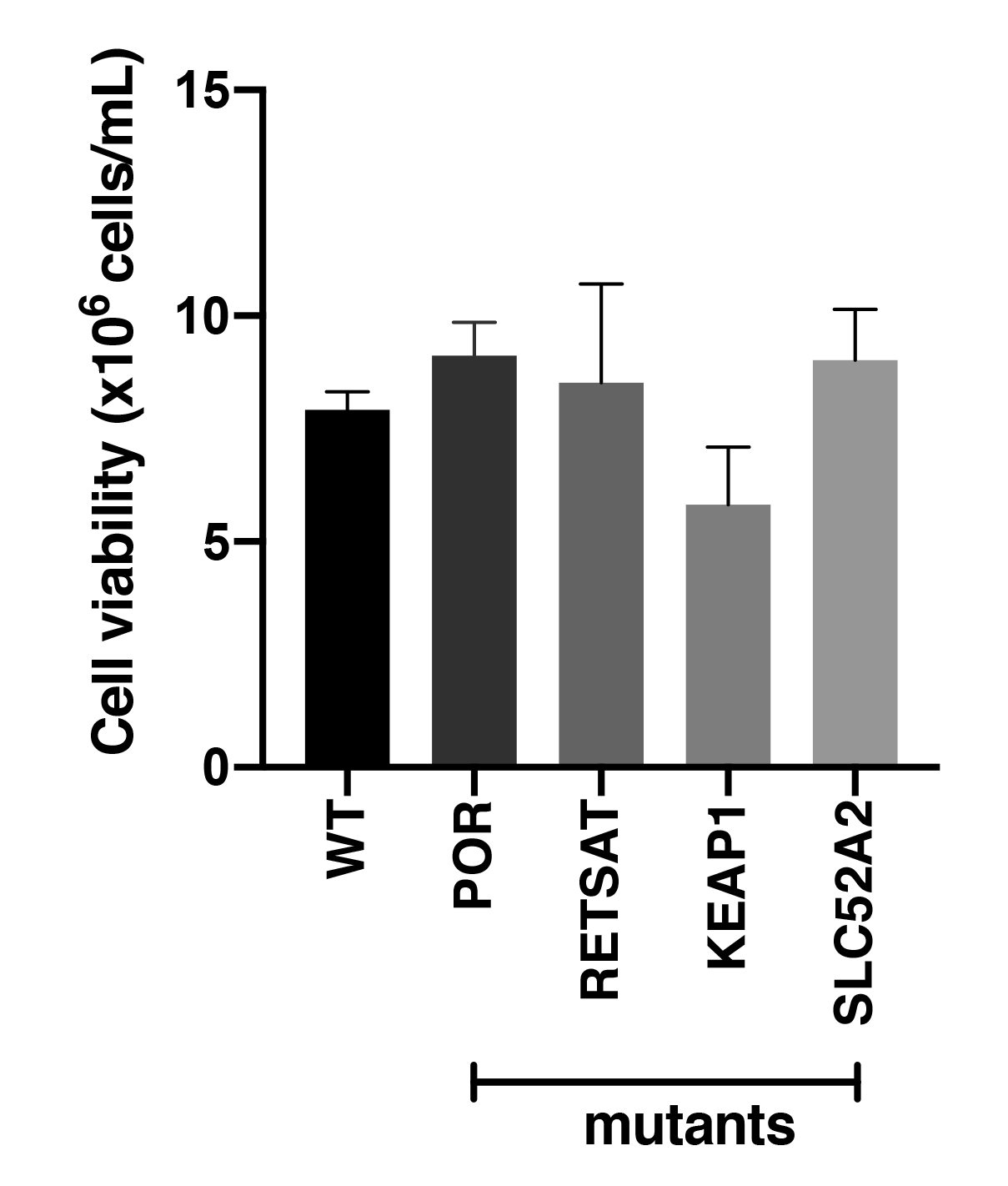

Supplement: FIG S3 [file mBio.01704-20-sf003.tif]

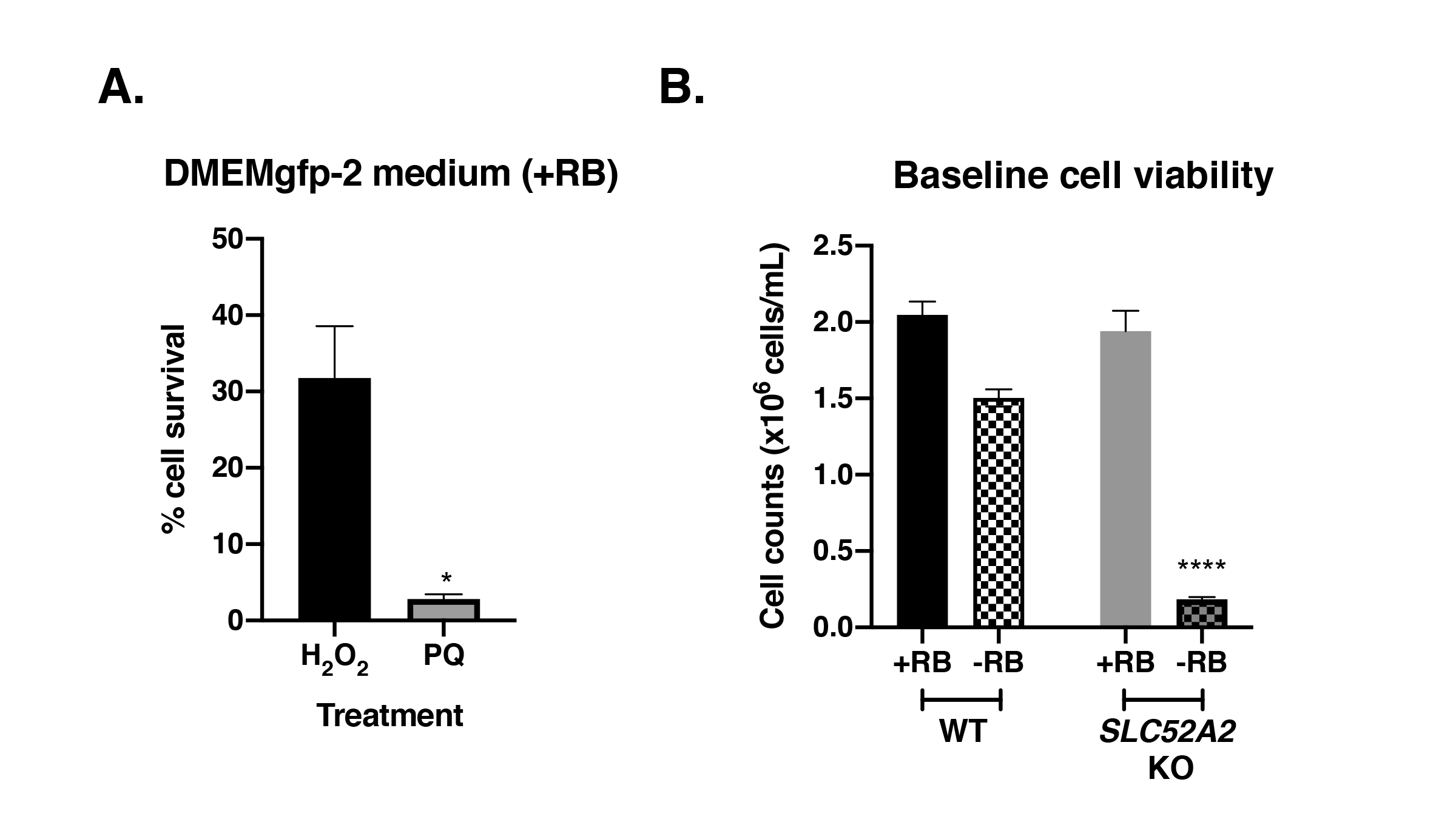

Supplement: FIG S4 [file mBio.01704-20-sf004.tif]

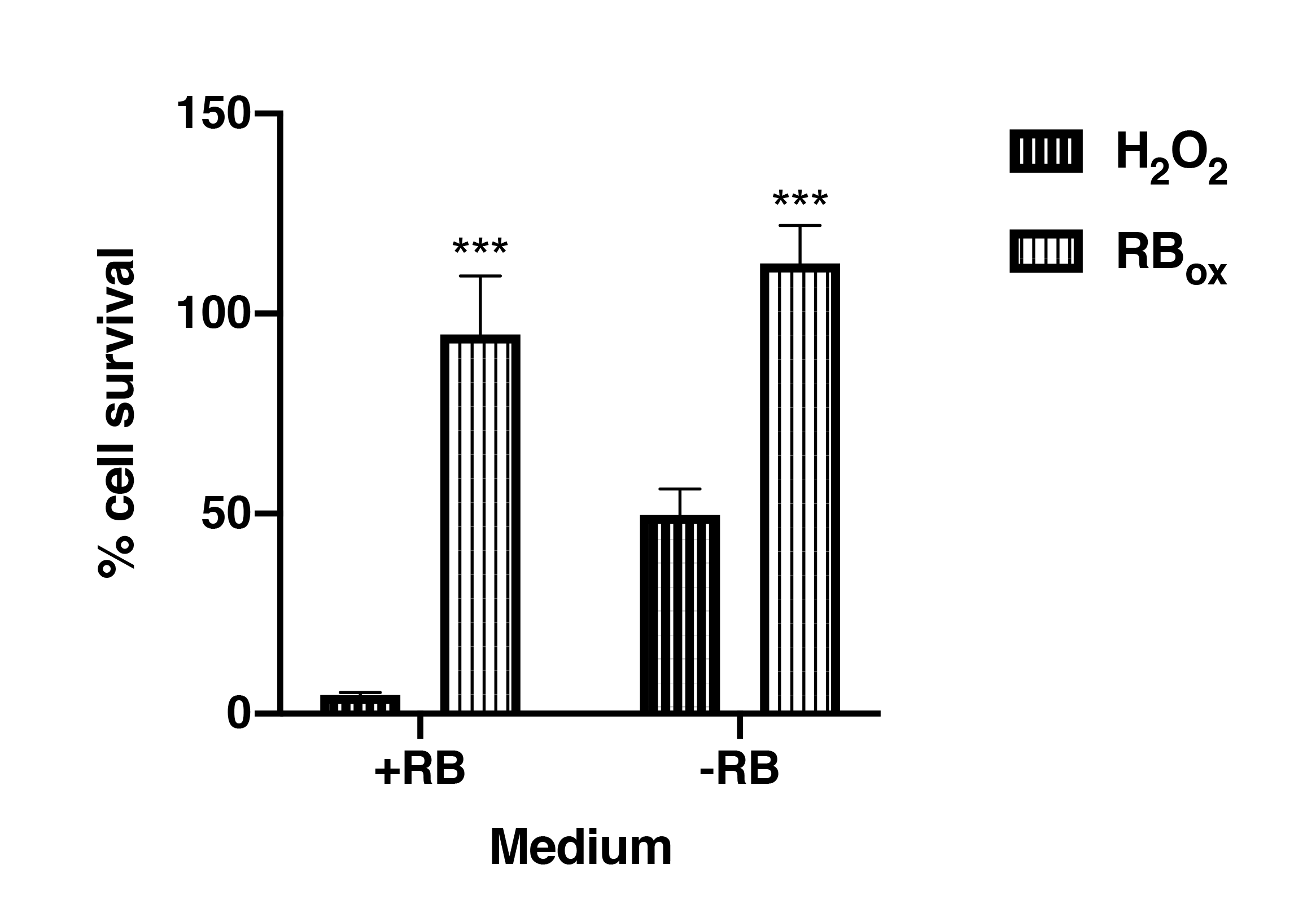

Supplement: FIG S5 [file mBio.01704-20-sf005.tif]

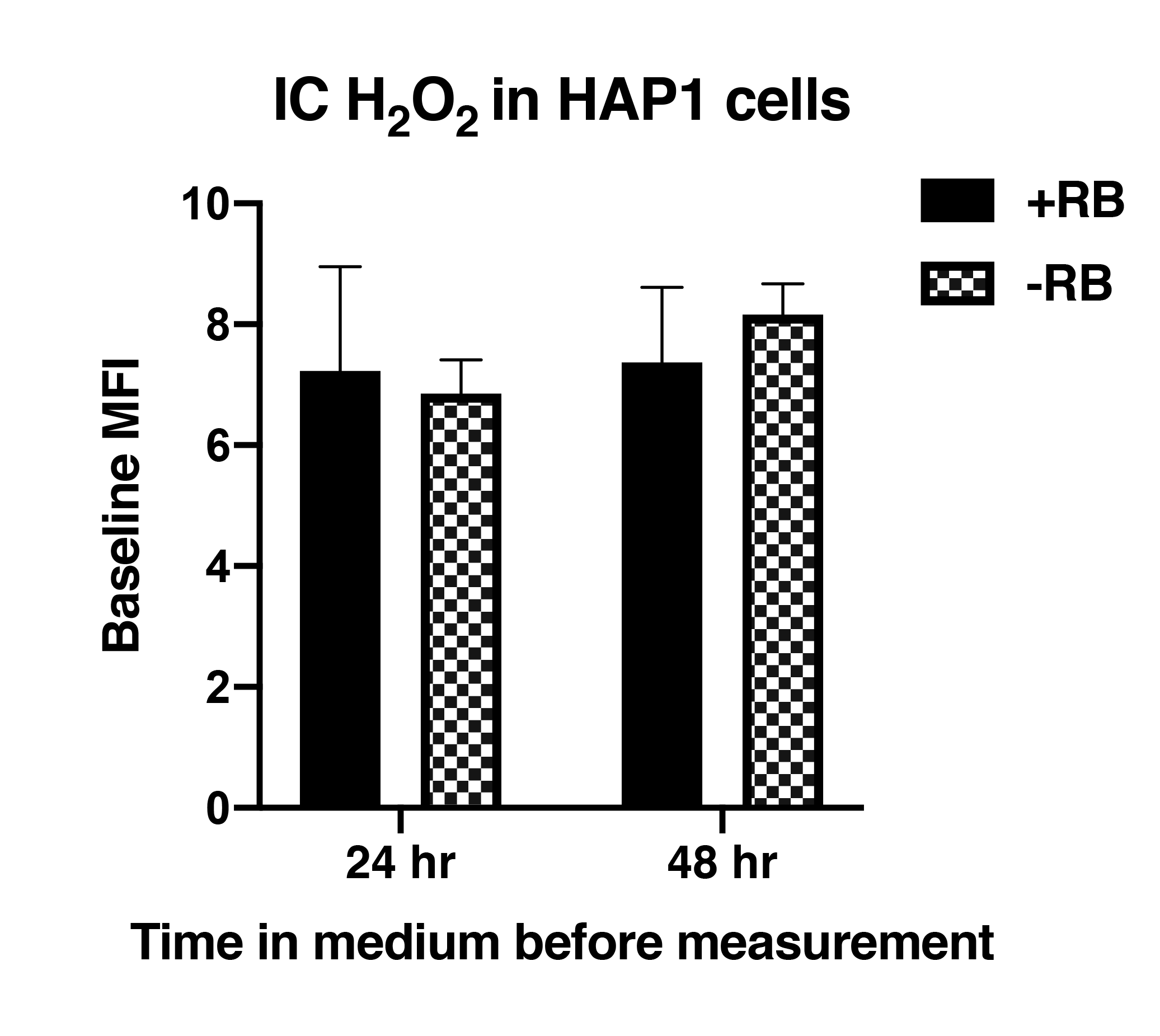

Supplement: FIG S6 [file mBio.01704-20-sf006.tif]

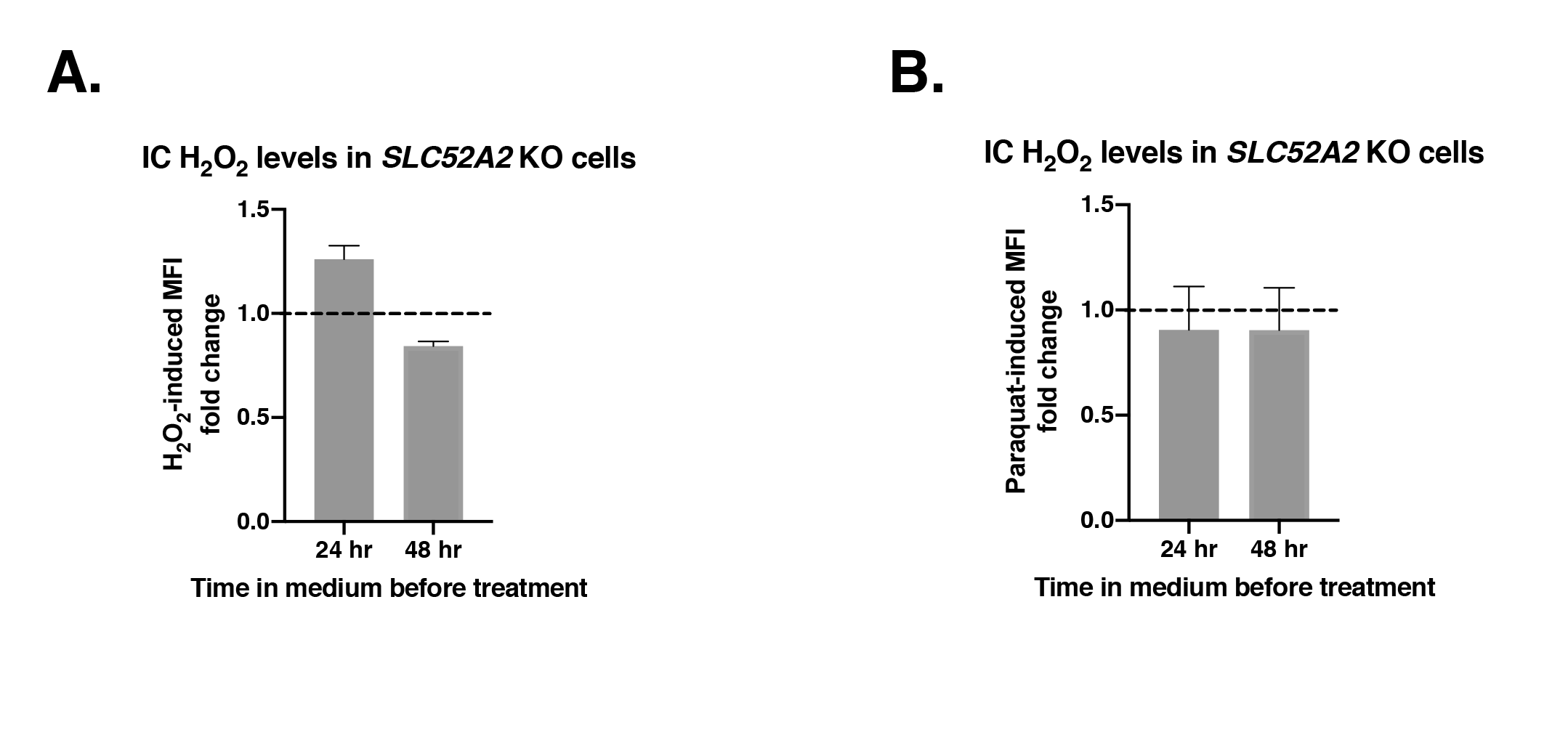

Supplement: FIG S7 [file mBio.01704-20-sf007.tif]

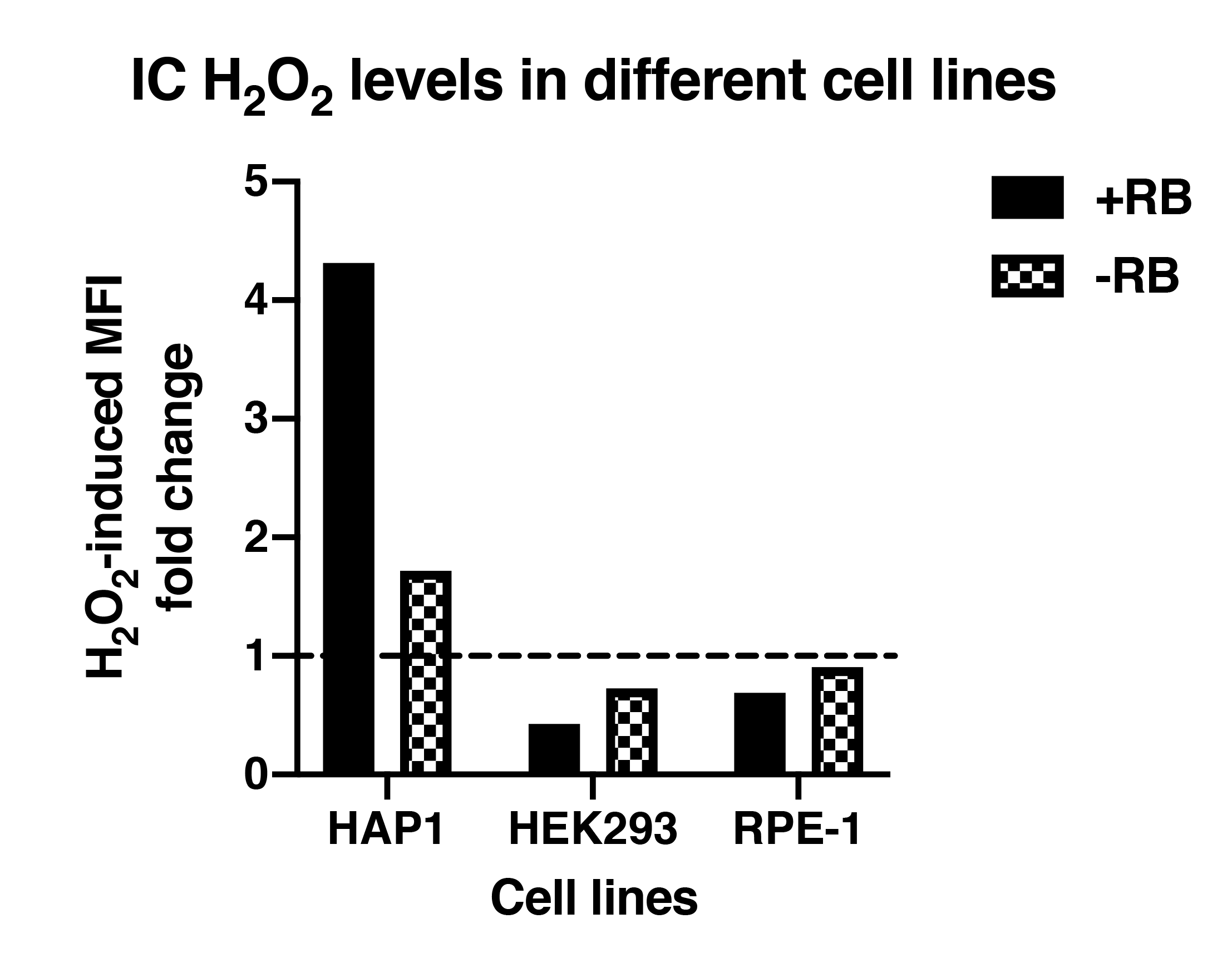

Supplement: FIG S8 [file mBio.01704-20-sf008.tif]
